# Evolutionary Dynamics of Wild Populations

**DOI:** 10.3390/genes12050778

**Published:** 2021-05-19

**Authors:** Delphine Legrand, Simon Blanchet

**Affiliations:** Station d’Ecologie Théorique et Expérimentale, UPR5321, CNRS, 09200 Moulis, France; simon.blanchet@lilo.org

Wild populations are facing rapid and sometimes extreme environmental changes that are currently exacerbated by pressing human activities. A major scientific endeavor is to reveal the evolutionary processes allowing wild populations to generate adaptive responses to these rapid and drastic environmental changes. In the recent decades, the accumulation of empirical data as well as the development of new theories and molecular tools have largely improved our ability to tackle such a major question. In particular, there is now growing evidence that evolutionary processes (gene flow, drift, mutation, and natural selection) interact in sometimes complex ways to shape the rapid responses of organisms to changing environments, and this can lead to unexpected feedback between evolutionary and ecological dynamics. These rapid responses are sustained by genetic determinants in addition to alternative inheritance systems, including those that are epigenetically controlled. Revealing these underlying molecular mechanisms of adaptation may change the way wild populations are managed and conserved.

This volume “Evolutionary dynamics of wild populations” synthesizes these novel and fascinating studies and provide a rare opportunity to generate a general overview of the ongoing projects tackling the difficult task of studying evolutionary dynamics in natural settings. Evaluating the adaptive potential of populations has long been a matter of interest in evolutionary biology, which recently became an important conservation criterion. By investigating the genome-wide molecular bases of insects’ adaptation to toxic host plants, Ferreira et al. [1] reveal footprint of selection in a key chemosensory gene family. They illustrate the importance of standing genetic variation for the emergence of different adaptive behavioral strategies. Working on nearly panmictic sea-water fish populations at a large spatial scale, Baltazar-Soares et al. [2] use a candidate gene approach to show that haplotypic frequencies correlate with thermal and oxygen conditions, suggesting adaptation to local environmental conditions despite a high degree of connectivity in this species. This fish might thus be able to follow environmental optima induced by global change. The fate of populations facing new environments might be influenced by many processes, including those linked to the landscape in which they live. For instance, Han et al. [3] suggest that the conservation status of the evergreen broad-leaved oak in East Asia should be evaluated based on the geographic localization of each population along their core-edge situation. Edge populations can sometimes harbor a similar global level of genetic diversity, but a unique allelic composition compared to that of core populations, suggesting that edge populations must be considered as independent conservation units. At the regional scale, Bal et al. [4] show that patterns of adaptive divergence in two sympatric stickleback species result from the interactive effects between species-specific characteristics and landscape features. They highlight similar levels of genetic diversity and neutral genetic differentiation between the two species, but different levels of morphological and adaptive genomic divergence. One of the two sympatric species systematically displayed a higher level of adaptive divergence, demonstrating the difficulty of extrapolating evolutionary dynamics from one species to another, even if they share a similar environment and a shared ancestry. At a lower spatial scale, Legrand et al. [5] show that the functioning of a natural metapopulation of butterflies does not rely on local dynamics of extinction/recolonization, but rather on a long adaptive history of the species to its local conditions. Especially, the fine-tuning of dispersal rates between populations according to local conditions favors the metapopulation equilibrium. However, other processes than those related to the landscape can strongly influence the fate of natural populations. Using reciprocal transplants between two recently founded populations of brown trout, Labonne et al. [6] show that sexual selection can have important effects on rapid evolutionary dynamics, even more than local adaptation or genetic rescue. By revealing the mechanisms sustaining patterns of genomic introgression in Brook Charr populations supplemented with the same domestic strain, Leitwein et al. [7] show little evidence of shared patterns of domestic ancestry between recipient populations. Patterns of introgression of recipient populations are rather dependent upon their initial genetic diversity, patterns of recombination and the stocking intensity. With the increasing availability of detailed genomic data at the chromosome level, conservation practices should increasingly benefit from the integration of such precise mechanisms in the management of populations, as they should benefit from the use of new tool to evaluate the identification of proper conservation units. Accordingly, Fargeot et al. [8] show that methylation profiles quantified in two sympatric freshwater fish populations better discriminate populations than do neutral genetic markers as those commonly used in conservation genetics studies. Although epigenetic profiles are expected to be strongly associated to environmental variation, they did not find evidence for this pattern in any of the two fish species, suggesting that higher mutation rate in epigenetic than genetic markers and neutral processes (drift) may sustain the higher discriminant ability of epigenetic markers. Methylation marks, as a molecular pathway for the regulation of gene expression, can be an important source of phenotypic variation in natural populations, notably sustaining rapid adaptation by phenotypic plasticity. Mouginot et al. [9] tested this hypothesis in the Snapdragon plant by investigating the link between DNA genome-wide patterns of methylation and degree of phenotypic plasticity in vegetative traits. They show strong epigenetic and phenotypic responses to change in light treatment. However, they surprisingly failed to detect a causal link between the epigenetic and phenotypic responses, pleading for further study on the proximal (molecular) mechanisms sustaining phenotypic variation commonly observed in wild populations. The microbiome can be another important source of phenotypic variation that can be adaptive and sometimes heritable. Bulteel et al. [10] propose to test the original hypothesis that tolerance to parasite in Daphnia could partly be explained by the microbiome carried by each individual. Although they failed to detect an important role of this proximal mechanism on their tolerance to parasites, they demonstrate a substantial role of the genetic background of hosts to both parasite tolerance and microbiome composition. Further studies are required to isolate the fitness benefits of carrying particular microbiomes.

This volume highlights the richness of studies focusing on the evolutionary dynamics of wild populations. It shows the diversity of organisms and approaches that can be used to reveal and understand empirical patterns, with—often but not always—the goal of improving the long-term conservation of wild populations. This diversity reflects the diversity of questions that occupy evolutionary biologists working in wild populations, which go from revealing their global (epi)genetic and phenotypic structure at different spatial and temporal scales to the search of the inherited bases of ecologically relevant phenotypic traits. This volume should be an important contribution to the field because firstly, papers selected in this issue provide answers to timely questions in evolutionary biology. Secondly, it proves that much has to be explored to understand the causes and consequences of evolutionary dynamics of wild populations, and hence that scientists still have to put effort into the study of wild populations.

## Data Availability

Not applicable.
